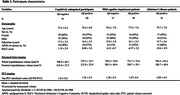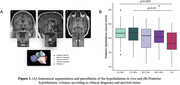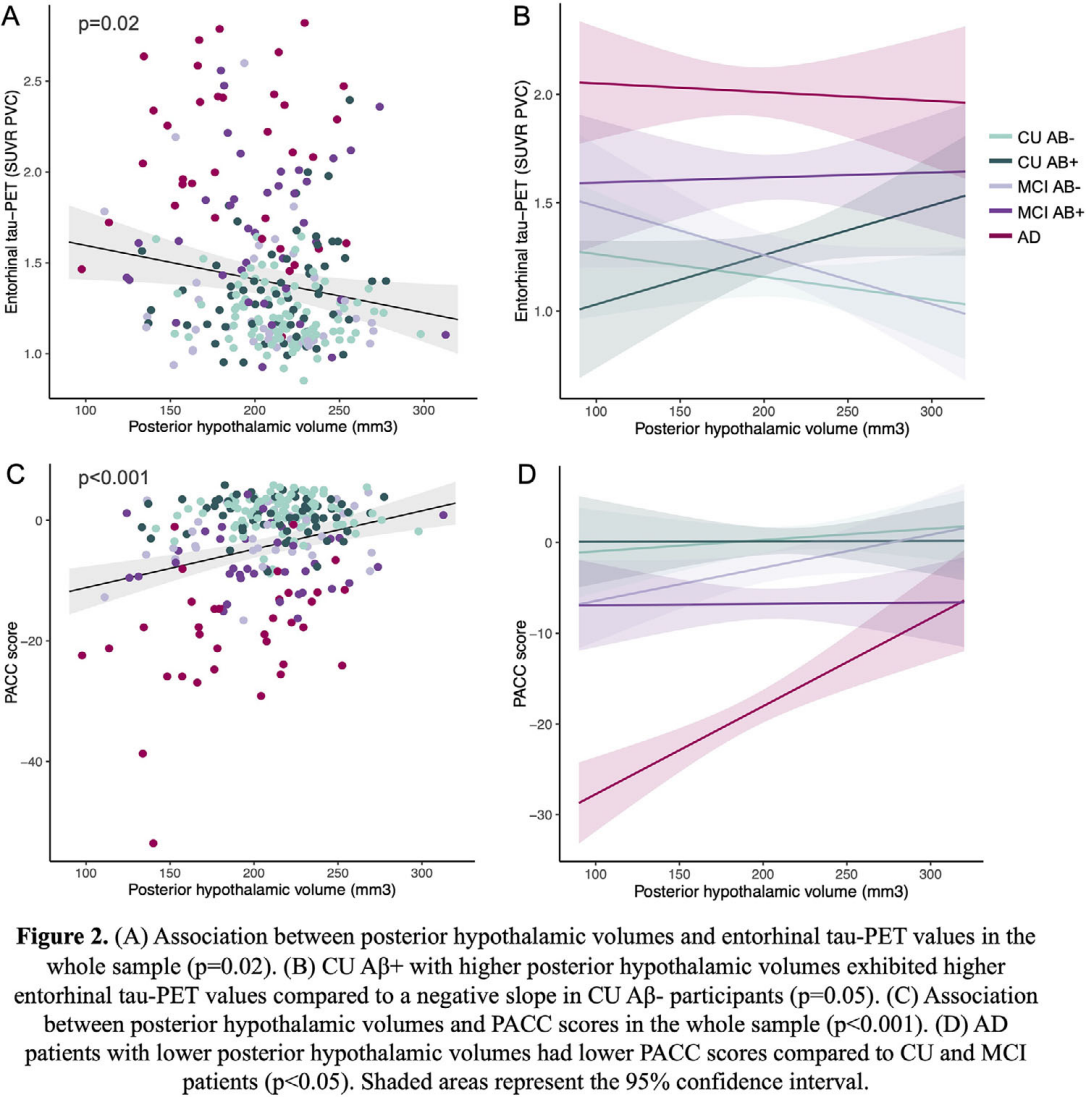# Association between in vivo structural integrity of the posterior hypothalamus, cognition and Alzheimer’s disease pathophysiology

**DOI:** 10.1002/alz.095329

**Published:** 2025-01-09

**Authors:** Marion Baillet, Tobey J. Betthauser, Eric Salmon, Heidi I.L. Jacobs

**Affiliations:** ^1^ Massachusetts General Hospital and Harvard Medical School, Boston, MA USA; ^2^ University of Wisconsin School of Medicine and Public Health, Madison, WI USA; ^3^ University of Liège, 4000 Belgium; ^4^ Maastricht University, Maastricht Netherlands

## Abstract

**Background:**

Abnormal tau accumulation is first observed in several neuromodulatory subcortical nuclei before its emergence in the allocortex, and long before the first deposits of amyloid‐beta (Aβ) plaques. Crucially, the posterior hypothalamus has been put forward as a critical site for Alzheimer’s disease (AD) pathogenesis but its role in AD‐related processes remains poorly investigated in humans. Here, we aimed to investigate whether the structural integrity of the posterior hypothalamus, as assessed in vivo, is associated with PET‐measured AD pathology and cognitive performance in cognitively unimpaired (CU) and impaired older individuals.

**Methods:**

151 CU individuals, 75 mild cognitive impairment (MCI) and 35 AD patients (mean ± SD age = 76.6 ± 6.7y., 137 women; **Table 1**) from the ADNI3 dataset were included. The volume of the posterior hypothalamus (corrected for intracranial volume) was derived from structural T1‐weighted images using a deep convolutional network algorithm (**Figure 1a**). ^[18F]^Flortaucipir‐PET was used to measure tau burden in the entorhinal cortex (standardized uptake volume ratio [SUVR], partial volume corrected values). Amyloid‐β (Aβ) positivity was determined with ^[18F]^Florbetaben‐ or ^[18F]^Florbetapir‐PET in a cortical summary region (SUVR values). Cognition was evaluated with the Preclinical Alzheimer’s Cognitive Composite (PACC). Analyses were adjusted for age, sex, and education.

**Results:**

AD patients exhibited lower posterior hypothalamic volume compared to CU Aβ‐ (p = 0.003) and CU Aβ+ participants (p = 0.01) but no significant difference was observed between CU and MCI patients nor between MCI and AD patients (**Figure 1b**). Linear regression models revealed that lower posterior hypothalamic volume was associated with higher entorhinal tau‐PET values (p = 0.02, **Figure 2a**). In addition, CU Aβ+ with higher posterior hypothalamic volume exhibited higher entorhinal tau‐PET values compared to a negative slope in CU Aβ‐ participants (p = 0.05, **Figure 2b**). Finally, lower posterior hypothalamic volume was related to lower PACC scores (p<0.001, **Figure 2c**), which was mainly driven by AD patients (p<0.05 for comparisons with CU and MCI, **Figure 2d**).

**Conclusions:**

Our findings indicate that the posterior hypothalamus is linked to disease‐related pathophysiological and cognitive changes. Future analyses will explore whether posterior hypothalamic volume is associated with inflammatory markers and changes in tau accumulation and cognitive performance over time.